# The incidence of radiologically verified community-acquired pneumonia requiring hospitalisation in adults living in southern Sweden, 2016-2018: a population-based study

**DOI:** 10.1186/s12879-025-10468-7

**Published:** 2025-01-17

**Authors:** Elisabeth Rünow, Frida Valeur, Gustav Torisson, Karin Hansen, Christian Theilacker, Kristian Riesbeck, Jonas Ahl

**Affiliations:** 1 Clinical Microbiology, Department of Translational Medicine, Faculty of Medicine, Lund University, SE21428 Malmö, Sweden; 2 Infectious Diseases, Department of Translational Medicine, Faculty of Medicine, Lund University, SE21428 Malmö, Sweden; 3 Vaccines Global Medical Development, Scientific and Clinical Affairs, Pfizer, Collegeville, PA USA; 4 Clinical Microbiology, Infection Control and Prevention, Laboratory Medicine Skåne, Lund, Sweden

**Keywords:** Age, CAP, Community acquired pneumonia, Epidemiology, Incidence, Mortality, *Streptococcus pneumoniae*, Vaccination

## Abstract

**Background:**

Community-acquired pneumonia (CAP) was one of the most common causes of death in the European Union in 2017. Severity and mortality of CAP increase with age and an aging European population will require increased planning for prevention, control, and management of CAP. The purpose of this study was to provide an updated population-based estimate of the incidence of CAP requiring hospitalization in Northern Europe.

**Method:**

We conducted a retrospective cohort study estimating the population-based incidence of CAP requiring hospitalization. Adults residing in Southern Sweden admitted between September 2016 and September 2018 with radiographically confirmed CAP and a primary discharge diagnosis consistent with pneumonia were identified by retrospective medical chart review. Incidence rates were stratified by age and sex.

**Results:**

We identified 1,575 episodes of CAP in 1,471 unique individuals, accounting for 45% of the total eligible patient population. The crude incidence rate of CAP requiring hospitalization was 259 (95% CI: 246–272) and age-standardized rate was 294 (95% CI: 280–309) per 100,000 person-years. Among those aged 80 years and older, hospitalization rate was 17 times higher vs those aged 18–64 years, yielding an IRR 17.4 (95% CI: 15.4–19.7). Males aged ≥ 80 years had a 57% increased risk of CAP requiring hospitalization compared to women ≥ 80 years, resulting in an IRR of 1.57 (95% CI: 1.33–1.85). The lowest in-hospital case-fatality risk was among the 18–64 years group 3.4% (*n* = 16), and highest among those ≥ 80 years 8.1% (*n* = 46).

**Conclusion:**

We found that the incidence and mortality of CAP requiring hospitalization in adults are considerable. Preventive measures are needed that target older adults and those at increased risk of CAP.

**Supplementary Information:**

The online version contains supplementary material available at 10.1186/s12879-025-10468-7.

## Introduction

Community-acquired pneumonia (CAP) is an infection of the lung parenchyma contracted outside the hospital setting. Respiratory infections are the third most common cause of death, causing an estimated 336,000 deaths, or 8% of all deaths, in the European Union in 2017 [1]. Among bacterial CAP, *Streptococcus pneumoniae* causes more mortality than all other etiologies combined; however, reports recent show a decreasing incidence [2]. Conjugated vaccines against *Haemophilus influenzae* type b (Hib) and pneumococci were implemented for all infants in Sweden in 1993 and 2009, respectively. This has probably changed the etiology of adult CAP in part due to herd effects [3–5].

The population in Sweden and many other European countries is aging, and the number of people aged 80 years and older is estimated to increase substantially until 2030 [6]. High age is associated with CAP severity and mortality, suggesting an increased CAP burden due to this demographic change [7, 8] that may lead to increase of hospital admissions, increased costs and potentially deaths [9]. The planning for prevention, control, and management of CAP therefore requires updated population-based incidence estimates.

Incidence studies of CAP can be performed by a prospective design using clinical symptoms and radiology for case definition or a retrospective design where diagnostic codes are used for case definition. International studies describing incidence are frequently reported, though studies from the Nordic countries are scarce. In a prospective incidence study performed by Bjarnason et al. during 2008–2009, the hospitalization rate was reported to be 200–300 adults/100,000 person-years (pyrs), whereas Sogaard and collaborators reported the hospitalization rate to be twice as high—400–600 adults /100,000 pyrs (study period 1997–2011) [10, 11].

Since there is no established international case definition for diagnosing CAP, diagnostic criteria for CAP vary depending on local traditions, the attending physician and hospital coding practices. Few studies in the Nordic countries have been performed using standardized codes to retrospectively identify cases of pneumonia among hospitalized patients, indicating a need for further investigation into the subject. The main aim of this study was to provide an updated population-based incidence estimate of CAP requiring hospitalization in Southern Sweden.

## Methods

### Overall study design

This retrospective cohort study complements the prospective investigation “Etiology of community acquired pneumonia in Skåne” (ECAPS). The method used in the present study was developed for the ECAPS study and follows the protocol and statistical analysis plan as described in the supplementary material.

### Setting

The catchment area was the municipalities of Malmö, Vellinge, and Svedala which is part of Skåne County in Southern Sweden (Figure S1 in Supplementary appendix). Skåne has 10 hospitals which together cover the entire catchment area. Vellinge and Svedala are two smaller rural municipalities close to Malmö. In Skåne, patient data is recorded electronically via the electronic medical record system that contains information on all admitted patients.

### Data sources

From the regional administrative database in Skåne, we retrieved data on adults aged ≥ 18 yrs, residing in the three municipalities Malmö, Svedala or Vellinge, that were admitted to any of the 10 hospitals in Skåne during the period 18 Sept 2016 to 18 Sept 2018 with a principal discharge diagnosis consistent with pneumonia. The ICD-10 diagnoses representing pneumonia included two ICD-10 diagnoses with COPD [12]. Our clinical experience is that patients with COPD are often inaccurately coded with a COPD exacerbation diagnosis when they seek inpatient care, even if pneumonia cannot be excluded. Therefore, we wanted to study how many patients with a COPD diagnosis that met the criteria for pneumonia.

### Case definition

A case of hospitalization due to CAP was defined when the following criteria were fulfilled:

1. Living in the catchment area on 18 Sept 2016 to 18 Sept 2018.
2. An ICD-10-SE discharge diagnosis code in the primary position consistent with pneumonia as specified in the supplementary material.
3. The presence of, e.g., pleural effusion, increased pulmonary density due to infection, and the presence of alveolar infiltrates (multilobar, lobar or segmental) with air bronchograms, as confirmed by two radiologists on duty. If consistent with the overall clinical presentation with the patient, pleural effusion was considered supportive of the pneumonia diagnosis in some cases.

The positive predictive value (PPV) of ICD-10 codes plus radiologic criteria was compared to a gold standard of ICD-10 codes, radiologic criteria plus clinical criteria. Since ICD-10 plus radiologic criteria had 99% accuracy, the case definition used for the final analysis was based on ICD-10 codes and radiological criteria only (supplementary material).

### Exclusion criteria

Patients were excluded if they (1) were hospitalized for ≥ 48 h in an in-patient facility (such as a community hospital), or (2) had been discharged from a hospital within 30 days prior to admission for pneumonia.

The same patient may have accounted for multiple episodes during the specified study period, provided they met enrolment criteria. Unique episodes were defined by an interval of ≥ 30 days between discharge and re-admission for pneumonia. These were manually reviewed to get a correct number of eligible cases.

### Risk factors for CAP and outcomes

Information about the age at admission, sex, and comorbidities were collected from electronic medical records. Patients were classified to different risk groups reflecting comorbidities. Classification is outlined in supplementary material. In addition, outcome (length-of-stay, case-fatality rate in hospital and ICU-admission) was noted.

### Statistics

The overall crude incidence rate for all-cause CAP was estimated by dividing the number of cases fulfilling the eligibility criteria, with the person-years at risk from the population data as the denominator. The data was based on the population of Skåne at the end of 2016 and at the end of 2017 according to the statistical database of Statistics Sweden [13]. The results are presented in text as a number with 95% CI per 100,000 pyrs.

In addition, the age-standardized incidence rate (ASR) was estimated using the direct standardization method, with the 2013 European standard population as a reference population [14]. The population was divided into subsets of 5 years strata. The reason for this was to increase comparability with other settings, as we knew that the population of Malmö is younger, and age is a major demographic risk factor [15]. Incidence rates were stratified by age [18–64, 65–79, ≥ 80 yrs] and sex. To compare incidence rates across these different strata, incidence rate ratios (IRRs) were used. Confidence intervals were estimated using the exact method.

### Sensitivity analyses

To increase the possibility of catching more misclassified pneumonia diagnoses, the database was queried once more with a pneumonia diagnosis in a non-primary position. Of these, we performed chart review in a sample of 5 patients for each month, resulting in 125 patients. The patients were sampled by date, and the first five patients registered for every month were chosen. The proportion of cases fulfilling the case definition with a pneumonia diagnosis in the non-primary position was extrapolated to the total number of hospitalizations with a pneumonia diagnosis in the nonprimary position. The codes COPD (J44.0 and J44.1) are not included in some studies [11, 16, 17]. To facilitate comparability among other studies we therefore present an analysis without these codes.

## Results

### Number of cases and overall incidence rate

**Fig. 1:**
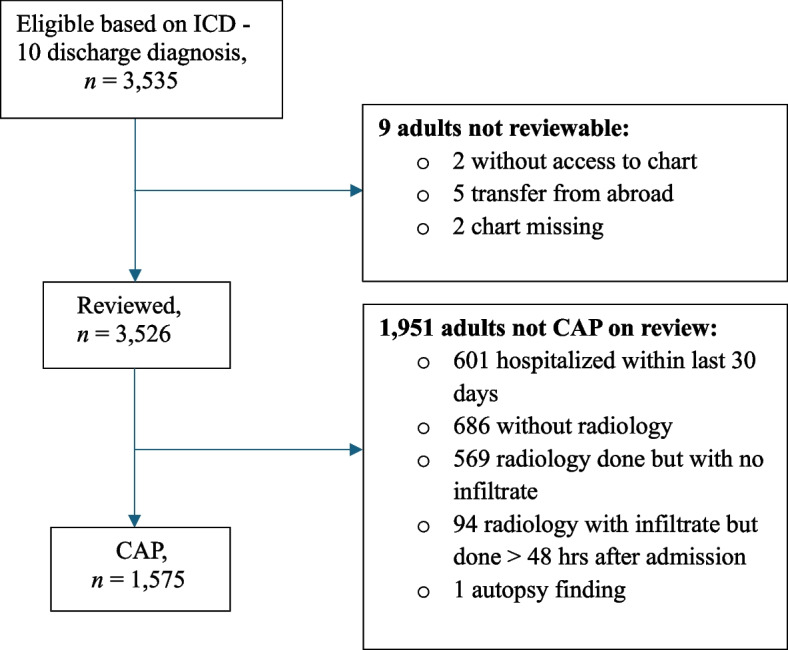
The flowchart represents eligibility and enrollment of patientes with community acquired pneumonia (CAP) in the catchment area during the study period

We identified 1,575 CAP episodes, representing 45% of all patients eligible in 1,471 unique individuals (Fig. 1). The most common reasons of not being considered CAP were: (1) missing X-ray, (2) patient hospitalized within last 30 days, (3) no radiological findings associated with pneumonia. Of 1,575 CAP episodes 1,564 (99.2%) had been hospitalized at the hospitals in Malmö, Lund and Trelleborg, which were closest to the three included communities. All hospitals in the region were included. The catchment area residents in Malmö, Svedala and Vellinge was 608,994. Thus, the crude incidence rate (IR) of CAP requiring hospitalization was 259 (95% CI: 246–272) per 100,000 pyrs. The age-standardized rate (ASR) was 294 (95% CI: 280–309) per 100,000 pyrs.

### Sensitivity analysis

1. In addition to the group with final diagnosis in primary position, 1,200 patients had a pneumonia diagnosis in a non-primary position. In a review of 125 randomly selected patients, 22 additional patients (17.6%) met our case definition of CAP with radiology and clinical symptoms indicating CAP. If this sample was extrapolated to all episodes with a pneumonia diagnosis in a non-primary position, this would result in a crude IR of 295, and an ASR of 337 per 100,000 pyrs.
2. In contrast, if CAP episodes with COPD diagnosis (J44.0 and J44.1) were excluded, the number of CAP episodes were 1,376 resulting in a crude IR of 226 and the ASR would be 257.

### Incidence by age group and sex differences

**Fig. 2:**
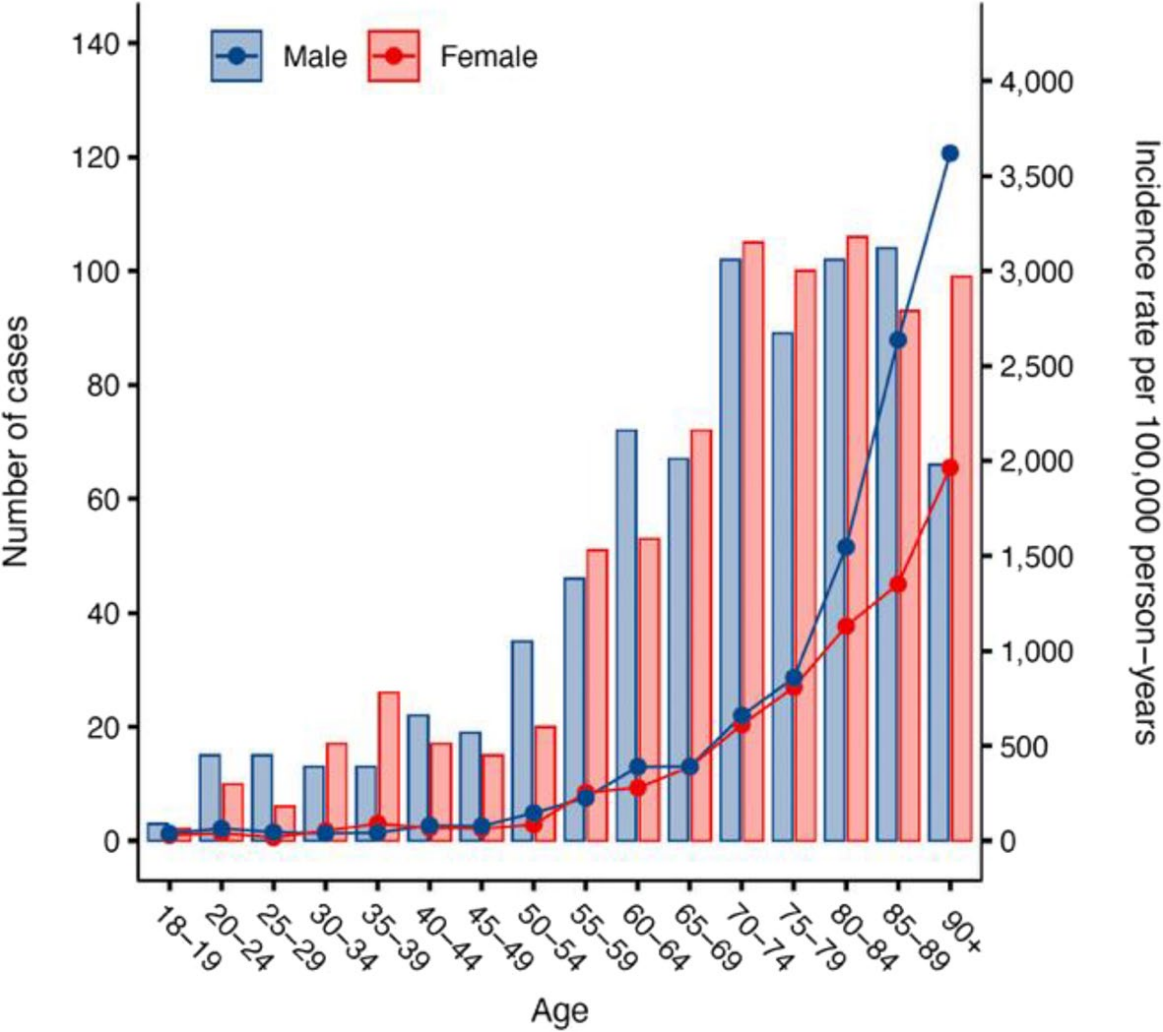
Number of cases and incidence rate by age and sex

The median age at CAP episode was 74 (interquartile range (IQR), 62–84 years). In the three age strata (18–64, 65–79, and ≥ 80 yrs), the crude incidence rates were 97, 588, and 1,693 per 100,000 pyrs, respectively. Among those aged ≥ 80 yrs, the incidence rate was 17-fold higher compared to those aged 18–64 yrs, resulting in an IRR of 17.4 (95% CI: 15.4–19.7). In total, 783 of 1,575 CAP episodes (50%) occurred in male patients ranging from 18 to aged ≥ 80 years. The proportion of males were almost consistent with increasing age representing 48.2% in the age group 65–79 yrs and 47.7% among men aged ≥ 80 yrs. The incidence rate in men and women were similar (262 *vs* 255) in the overall study population, resulting in an IRR of 1.03 (95% CI: 0.93–1.14) but differed in adults aged ≥ 80 yrs, where IR among men was higher (IR of 2,201) than in women (IR 1,399), resulting in an IRR of 1.57 (95% CI: 1.33–1.85) (Fig. 2).

In total, 1,201 patients (76%) had at least one comorbidity, with cardiovascular disease, chronic obstructive pulmonary disorder (COPD) and diabetes being the most common ones. Comorbidities were differently distributed across age strata, with solid organ transplants and immunosuppression most frequent in the youngest group, COPD, diabetes, and malignancies in the 65–79 yrs group and cardiovascular disease and heart failure in the oldest group (Table 1).

**Table 1:** Comorbidities and outcome by age categories

**Age group (yrs)**	**18–64**	**65–79**	** ≥ 80**
Patients (*n*)	470	535	570
Male sex (*n* [%])	253 (53.8)	258 (48.2)	272 (47.7)
**Comorbidities** (*n*, [%])			
Any comorbidity	297 (63.2)	439 (82.1)	465 (.6)
COPD^a^	50 (10.7)	191 (35.7)	130 (22.8)
Asthma	28 (6.0)	35 (6.5)	24 (4.2)
Heart failure	22 (4.7)	66 (12.3)	129 (22.6)
Peripheral vascular disease	66 (14.0)	202 (37.8)	305 (53.5)
Diabetes	77 (16.4)	121 (22.7)	93 (16.3)
Liver disease	22 (4.7)	17 (3.2)	0 (0.0)
Immunosuppression	80 (17.0)	74 (13.8)	40 (7.0)
CKD^a^	19 (4.0)	57 (10.7)	70 (12.3)
HIV^a^	9 (1.9)	0 (0)	0 (0)
AIDS^a^	0 (0.0)	0 (0)	0 (0)
Malignancy	31 (6.6)	79 (14.8)	53 (9.3)
Hematological disease	11 (2.4)	30 (5.6)	21 (3.7)
Transplanted organ	19 (4.0)	9 (1.7)	0 (0)
**Risk group** (*n*, [%])			
At risk^b^	129 (27.4)	256 (47.9)	305 (53.5)
High risk^b^	109 (23.2)	170 (31.8)	157 (27.5)
Low risk^b^	232 (49.4)	109 (20.4)	108 (18.9)
**Outcomes**			
Deceased (*n*, [%])	16 (3.4)	25 (4.7)	46 (8.1)
ICU^a^ (*n*, [%])	31 (6.6)	23 (4.3)	3 (0.5)
LOS^a^—days (median [IQR])	4.50 [3.00, 7.00]	5.00 [3.00, 9.00]	6.00 [4.00, 9.00]

^a^*Abbreviations*: *AIDS* acquired immunodeficiency syndrome, *ICU* intensive care unit, *CKD* chronic kidney disease, *COPD* chronic obstructive pulmonary disease, *HIV* human immunodeficiency virus, *LOS* length of stay

^b^Risk groups specified in supplementary appendix

In total, 87 patients died during the hospitalization, resulting in a case-fatality rate of 5.5%. In the group aged ≥ 80 years, the case-fatality risk was 8.1% (Table 1). The median length of stay was 5 days (IQR 3–9), with a total of 11,464 hospital nights, equivalent to 1,882 per 100,000 pyrs. As the oldest patient group also had longer length of stay (LOS), the age difference in hospital utilization was even more pronounced than for hospitalizations, with a rate of 12,418 hospital nights per 100,000 pyrs for the group 80 ≥ yrs vs 667 hospital nights per 100,000 pyrs in the group aged 18–64 yrs, resulting in an IRR of 18.62.

### ICD-10 diagnoses and diagnostic validity

The diagnoses, J18.9 (pneumonia, not otherwise specified), J15.9 (unspecified bacterial pneumonia), and J44.1 (COPD with acute exacerbation) accounted for 82.3% of the pneumonia diagnoses in our study cohort (Table 2). Of the original 3,526 CAP episodes, COPD (diagnostic codes J44.0 or J44.1) contributed 1,084 (31%) of all cases. Of those, 199 / 1,084 (18%) had radiological findings of pneumonia. Diagnoses with a specific etiology, such as Legionnaire’s disease or pneumonia due to *S. pneumoniae* had higher positive predictive value when matching diagnostic codes with radiographic findings (Table 3).

**Table 2:** Most frequent ICD-10 diagnosis in CAP cases

ICD	Diagnosis	*n*	%
J189	Pneumonia, NOS^a^	817	51.9
J159	Bacterial pneumonia, NOS	288	18.3
J441	COPD^a^ with acute exacerbation	191	12.1
J690	Pneumonitis due to inhalation of food and vomit	65	4.1
J100	Influenza with pneumonia	45	2.9
J139	Pneumonia due to *S. pneumoniae*	38	2.4
J149	Pneumonia due to *H. influenzae*	24	1.5
J869	Pyothorax without fistula	17	1.1
A481	Legionnaire´s disease	16	1.0
J440	COPD with acute lower respiratory infection	8	0.5
Other	Other	66	4.2

^a^*Abbreviations*: *NOS* not otherwise specified, *COPD* chronic obstructive pulmonary disease

**Table 3:** Positive predictive value of ICD-10 diagnoses in CAP

ICD	Diagnosis	Number with ICD -code	Number with adjudicated CAP^a^	Positive predictive value of ICD-10 codes (%)
A481	Legionnaire´s disease	19	16	84
J869	Pyothorax without fistula	23	17	74
J149	Pneumonia due to *H. influenzae*	34	24	71
J139	Pneumonia due to *S. pneumoniae*	54	38	70
J159	Bacterial pneumonia, NOS^b^	501	288	57
J690	Pneumonitis due to inhalation of food and vomit	115	65	57
J189	Pneumonia, NOS^b^	1466	817	56
J100	Influenza with pneumonia	99	45	45
J440	COPD with acute lower respiratory infection	32	8	25
J441	COPD with acute exacerbation	1054	191	18
Other	Other	130	66	51

^a^Adjudicated CAP = Cases with ICD-10 code for pneumonia and a positive X-ray after manual chart review

^b^*Abbreviations*: *NOS* not otherwise specified, *COPD* chronic obstructive pulmonary disease

## Discussion

In this retrospective population-based study, we found that the annual incidence rate of radiologically adjudicated CAP requiring hospitalization was ranging from 259 to 337 cases per 100,000 pyrs, depending on the use of age-standardization and the inclusion of ICD codes in non-primary positions. Hospital utilization for CAP was 19 times higher in the age group ≥ 80 yrs compared to 18–64 yrs (IRR 18.6) and in this age group the case-fatality risk was 8.1%.

Our crude incidence rate of 259 cases per 100,000 pyrs is in line with the prospective studies conducted by Jain et al*.* and Marston et al*.* with 248 and 267 per 100,000 pyrs [7, 18]. Other studies indicate higher figures demonstrating almost identical incidence numbers of 649 and 645 per 100,000 pyrs [19, 20]. The comparison of studies is complicated by the lack of a reference standard for diagnosis, different study designs and settings, with variations in local routine, diagnostic accuracy, and access to hospital care. In addition, population demographics are important and may complicate comparisons across settings. Our catchment area has a population younger than national average as indicated by the increase in IR from 259 to 294 upon age-standardization, emphasizing the need to provide standardized estimates. Including pneumonia diagnoses in non-primary positions resulted in an increase in crude IR from 259 to 293 cases per 100,000 pyrs and in ASR from 294 to 337. On the other hand, only 18% of the reviewed cases with pneumonia diagnoses in non-primary positions fulfilled radiology criteria, suggesting that non-primary codes should not be included per se but that reviews of diagnostic accuracy are necessary in this group.

A significant number of patients (*n* = 601) had to be excluded since they were hospitalized in the last 30 days, highlighting the burden of HAP. In line with previous studies, we demonstrated that older individuals had a significantly increased hospital utilization rate, especially after 80 yrs of age [7, 17, 19, 21]. When combining age and sex there was no difference in ages 18–79 but males aged ≥ 80 yrs had a more than 50% higher IR compared to women of the same age. This finding, that has been previously described, may have implications on focused preventive strategies, as this age group is expected to increase substantially [22]. Most included patients also had comorbidities associated with risk of CAP. These comorbidities varied with age; among the population aged 18 to 64 yrs, immunocompromising conditions and organ transplantation were dominating, emphasizing the need for targeted prevention in this group. In the group aged over 80 yrs, co-morbidities including cardiovascular conditions, heart failure and COPD were the most common.

The overall in-hospital case-fatality risk was 5.5%, which is higher than the 3.7% in the prospective ECAPS study [23]. Other prospective studies have reported in hospital mortality ranging between 2.0%−6.5% and in retrospective studies between 7.9% −17.2% [7, 16, 19, 24]. The differences in mortality rate could be explained by the fact that the frailest or most severely diseased patients may not be enrolled in prospective studies due to exclusion criteria which is demonstrated by the ECAPS study where only 47% of eligible patients were included [23]. We included codes representing COPD with acute exacerbation (ICD-10 codes J44.0 or J44.1) in our case-finding. Among these, 18% had CAP according to our definition. Nevertheless, this was a large diagnostic group and among the patients with radiology-verified CAP, one third of CAP was misclassified as COPD exacerbation in our study. Previous studies of ICD-10 discharge diagnosis codes usually do not include COPD codes as an indicator of CAP, which could lead to an underestimation of CAP incidence. [7, 11, 16, 17, 19]. However, the low specificity suggests that these codes should not be included without review of radiology.

In our study, 45% of patients diagnosed with pneumonia or COPD exacerbation fulfilled the CAP criteria after review, indicating that these ICD codes alone may overestimate CAP incidence. Compared to previous studies of diagnostic accuracy, this proportion was lower, but our case definition also included J44 as a non-pneumonia ICD-10 code [25]. Among the most common reasons for not meeting CAP criteria was lack of infiltrates on radiology. Adding radiology as a diagnostic CAP criterion could underestimate CAP incidence since the sensitivity of chest X-ray is only 44 to 77% and is often negative in the first couple of days in older patients with pneumonia [26]. Moving from the traditional chest X-ray to CT could increase sensitivity; a study by Claessen *et al*. found that a CT scan improved diagnostic accuracy in suspected CAP in 59% of cases [27]. In addition, the lack of reference standard enables the diagnosis of pneumonia without an evident finding on radiology and with increasing cost-pressure any attempts are made to reduce diagnostic test that do not impact patients’ management. Furthermore, as mentioned above, the addition of COPD codes, with only 18% representing CAP cases after review, reduced specificity.

The strengths of this study are that it was population-based, and that all episodes with an ICD diagnosis of pneumonia were confirmed by a thorough review of radiology findings. The retrospective design made it possible to include a full population sample, including frail patients, or those unable to give consent, a patient group that are often excluded from prospective studies. 99.2% of CAP episodes had been hospitalized at the three closest communities and since we included all hospitals in the region it is unlikely that patients seek medical care outside the region. In addition, the review of radiology findings justified the inclusion of COPD diagnoses, which enabled the identification of a subset of CAP patients often missed in previous studies. Limitations also exist. The retrospective study design is reliant on the treating physicians´ assessments, and documentation, with hospital discharge diagnoses being dependent on local routines. As stated above, patients treated for pneumonia without radiologic assessment or findings were not included as CAP cases in our analysis. Sweden has one of the lowest numbers of hospital beds per capita (2.1 per 1,000 inhabitants) as compared to other countries in Europe limiting the external validity [28]. This may entail a higher hospital admission threshold, with more CAP patients being treated in ambulatory care and consequently a lower hospitalization rate of CAP, compared to settings with more hospital beds per capita.

## Implications of findings

Our results highlight the lack of an established reference standard to define CAP, complicating the comparison of CAP epidemiology across settings. The use of age-standardization and diagnoses in non-primary positions as well as the selection of ICD diagnoses representing pneumonia have all been inconsistent in previous studies. This also makes the evaluation of the effect of additional interventions challenging. From a public health perspective, our results indicate a substantial burden of CAP. This burden is likely to increase due to the expected demographic change, with an increasing proportion of the population being aged over 80 yrs. We found an especially high hospital utilization with CAP in this group, highlighting the need for targeted preventive measures. After this study was performed, national vaccine strategies for risk groups and/or the elderly, targeting respiratory pathogens, including *S. pneumoniae*, COVID-19, and Respiratory Syncytial Virus (RSV) have been updated or added. Vaccine coverage in the prospective ECAPS study were only 15 and 49% with pneumococcal or influenza vaccines, respectively [23]. Therefore, we believe that further improvement and implementation of the existing vaccination program should be emphasized. However, cost–benefit needs to be evaluated to clarify if a more targeted vaccination program should be launched for these groups. Other interventions, such as smoking cessation and improved oral care have also been described as an important measure to prevent pneumonia and need to be addressed in risk groups [29]. Data obtained in the present study could potentially also be used to define specific target groups to add preventive measures.

## Conclusion

In conclusion, this study highlights the considerable burden of CAP, especially among the oldest population. With the expected demographic change, this burden is likely to increase, emphasizing the need to optimize preventive measures.

## Supplementary Information

Supplementary Material 1.

Supplementary Material 2.

## Data Availability

The dataset analysed during the current study is not publicly available due to privacy concerns but is available from the corresponding author on reasonable request. Such requests will need ethical approval as well as permission from the data protection officer at Skåne university hospital.
