# Supplementary material for: The incidence of radiologically verified community-acquired pneumonia requiring hospitalisation in adults living in southern Sweden, 2016-2018: a population-based study
Source: BMC Infect Dis. 2025 Jan 17;25:80. doi: 10.1186/s12879-025-10468-7 (PMC11742510; doi:10.1186/s12879-025-10468-7)
Supplement: Supplementary file 1 — Supplementary Material 1. [file 12879_2025_10468_MOESM1_ESM.pdf]

Malmö 2019-03-01

Protocol and statistical Analysis Plan – Incidence calculations from retrospective cohort

Authors: Elisabeth Rünow and Gustav Torisson

Study background and rationale.

Community-acquired pneumonia (CAP) is an infection of the lung parenchyma contracted outside of the hospital setting and continues to be a major cause of morbidity and mortality worldwide. Pneumonia and influenza combined are the 8th most common cause of death in the United States (US) and a leading cause of death from infectious disease (1).

In Sweden the most common etiology of pneumonia that require hospital care is S. pneumoniae followed by H. influenzae and respiratory viruses (2, 3). The prevalence of S. pneumoniae in CAP, particularly non-bacteremic CAP, is likely underestimated due to a lack of sensitive and specific assays. Certain demographics, such as age above 65 years, male gender, and nursing home residence are described as risk factors associated with moderate to severe CAP and/or increased probability of death (4). Despite difficulties to identify etiologic agents associated with CAP, S. pneumoniae was estimated to cause nearly 600,000 cases of pneumococcal pneumonia in adults ≥18 years of age in the US in 2004. Approximately 50% of the cases occurred in patients aged ≥ 65 years. Hospitalization also occurred more frequently in older populations.

The incidence rate of CAP requiring hospital admission can be estimated prospectively or retrospectively. A prospective approach usually implicates the inclusion criteria of respiratory tract symptoms and a confirming X-ray for diagnosis. A retrospective approach assesses ICD-codes (International Statistical Classification of Diseases and Related Health Problems)

and includes patients diagnosed with pneumonia when discharged from hospital (5-9)

Good quality data on incidence of pneumonia are scarce in the Nordic countries. A prospective study in Finland by Jokinen et al (10) was done in the 1990s estimating the incidence of CAP including all ages to 116 per 100 000 population per year. Sweden is the country in OECD with fewest acute hospital beds per capita, with 2.1 per 1000 inhabitants in 2018. The rate of hospital beds per capita has decreased by 43% in the last 20 years, primarily due to a major change towards outpatient management in all medical fields. Thus, the threshold for hospital admission has likely been raised for many acute conditions, including pneumonia. This also affects comparability with studies from the 1990s.

Since 2009 has Pneumococcal conjugated vaccine (PCV) been used in the child immunization program in Sweden where the immunization coverage in Sweden is 97% for children born in 2015 (11). Many studies describe the herd effects and replacement with nonvaccine serotypes This also emphasize the need for a new incidence study.

In Sweden, the prospective study ECAPS (Etiology of CAP in Sweden) was performed at Skåne University hospital Malmö between 16th of September 2016 and 16th of September 2018. The study aimed to determine the full distribution of all *S.pneumoniae* serotypes among adults with CAP and also to estimate the incidence rate of CAP. Adults ≥18 years of age living in Malmö, Svedala, and Vellinge who, at the emergency room, presented signs and symptoms of CAP, and had radiographic evidence of pneumonia were prospectively identified and enrolled. The prospective inclusion rate was however not complete and to get a more accurate hospitalizations rate of pneumonia, a complementary retrospective study was performed during 2020 to estimate the number of hospitalized CAP during 2016-2018.

Research Question(s)

1. What is the incidence rate of all-cause, pneumococcal CAP in hospitalized patients aged ≥18 years in a well described area in Southern Sweden during 2016-2018?
2. What is the incidence rate of all-cause CAP stratified by age, sex and risk strata based on comorbidities?
3. Which comorbidities are present in patients hospitalized with CAP?
4. What is the outcome of all-cause CAP?
5. Among study-eligible patients, which are most frequent ICD-10 pneumonia diagnoses codes occurring in the primary position?
6. How is specificity of ICD-10 diagnoses in CAP is related to etiologic and radiographic findings?

Endpoints

Primary endpoint:

- Overall incidence rate per 100.000 person-years of CAP among hospitalized adults living in the study catchment area.

Secondary endpoints:

1. In patients with all-cause CAP:
    - incidence rate by age (strata: 18-64, 65-79, 80+)
    - incidence rate by sex
    - incidence rate by risk stratification (low risk, at risk, high risk)
2. comorbidities in CAP patients (specific and grouped into low risk, at risk and high risk)
3. outcome of all-cause, pneumococcal CAP (length-of-stay, ICU, in-hospital mortality)

4. Among study-eligible patients, which are most frequent ICD-10 pneumonia diagnoses codes occurring in the primary position.
5. Among study-eligible patients how is specificity of ICD-10 diagnoses in CAP related to etiologic and radiographic findings.

Overall study design

We conducted a retrospective study of patients ≥ 18 years living in Malmö, Svedala, or Vellinge who were hospitalized with community-acquired pneumonia in Skåne between 18th of September 2016 to 18th September 2018. We went through every patient's medical chart and examined how many patients met the eligibility criteria of the OSPIS study

Incidence rate was calculated, also in relation to age, gender, and season.

Setting

Sweden is divided into 21 regions where Skåne is the southernmost with a population of just over 1,3 million people at the turn of the year 2019/2020. Just over half of Skåne´s population lives in southwestern Skåne centered at Malmö, Lund and Helsingborg. Malmö is the third-largest city in Sweden, with a population about 348 000 persons where 48% are under the age of 35. Approximately one-third of the residents in Malmö were born in another country, compared to approximately one-fourth in the other two big cities in Sweden, Göteborg, and Stockholm (4). Vellinge and Svedala are two smaller rural municipalities with an average age of 43 and 39 years respectively and the majority of the population is born in Sweden, 90.2% and 88.4% respectively. (12,13)

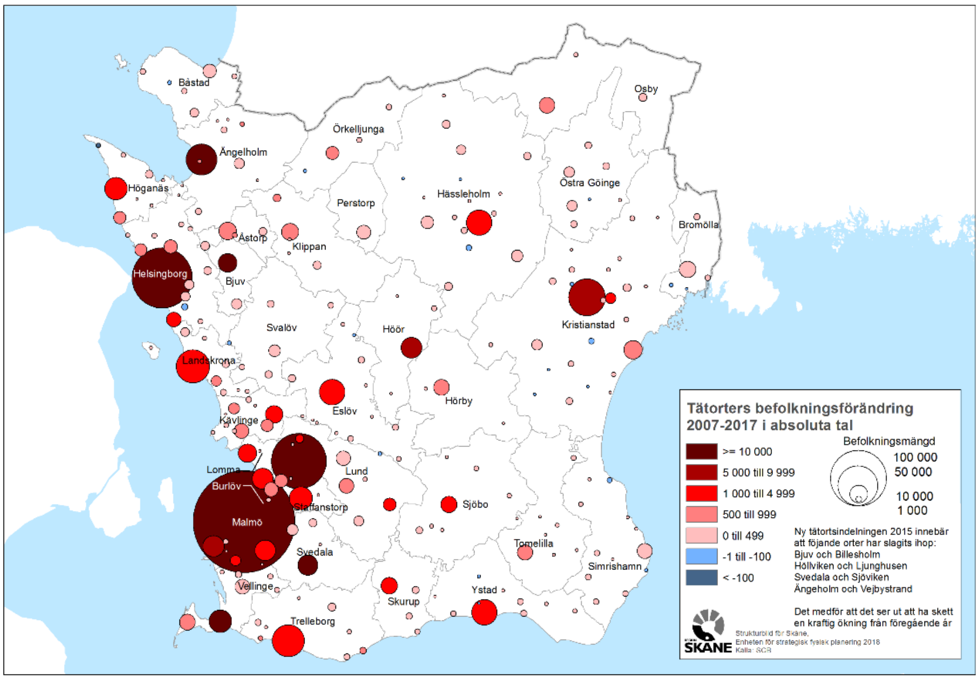

Figure 1. Geography and population distribution of the Skåne region.

Region Skåne is the regional municipality in Skåne County. The region is primarily responsible for health and medical care and can offer citizens medical care at 10 hospitals around the county: (Skåne University hospital [SUS] Malmö and Lund, Trelleborg, Ystad, Kristianstad, Hässleholm, Helsingborg, Ängelholm Landskrona, and Simrishamn).

Region Skåne can offer the inhabitants almost all aspects of modern healthcare and therefore the residents do not have to travel outside the county to receive adequate healthcare. Most of Skåne´s ten hospitals are located in the western part of Skåne, including the university hospital (SUS), which is situated in Malmö and Lund. Skåne´s university hospital is a tertiary referral hospital with 600 beds and the only hospital located in Malmö. In the Skåne region, patient data is recorded electronically via the Melior medical record system that provides support for both outpatient and inpatient care. Melior contain information on all patients who have been admitted to hospital, together with information about the primary and secondary causes of each episode of inpatient care (with diagnosis coded using ICD-10 international

classification of diseases, 10th revision), type of admission, procedure performed, length of stay, and discharge status.

Radiology at Skåne University Hospital

At Skåne university hospital in Malmö, all X-rays are taken digitally and transferred to the image review system PACS (picture archiving and communications system), which every clinician in the hospital has access to. All X-rays are analyzed twice where the second radiologist analyzing the X-ray must be senior and is the one who finally signs the X-ray report.

Definition of incident case

A patient was defined as an incident case / having CAP when he/she fulfilled the criteria of:

1. An ICD-10-SE discharge diagnosis code in the primary position, consistent with pneumonia, as specified below.
2. Presence of a new pulmonary infiltrate (radiographic finding that is consistent with pneumonia) at the time of hospitalization, defined by the clinical radiologist on duty.
3. Clinical symptoms. Two of the following: Fever (oral temperature; 38°C or tympanic temperature (38.5°C), hypothermia (35.5°C), chills or rigors, pleuritic chest pain, cough, sputum production, dyspnea, tachypnea (respiratory rate >20/ min), malaise or abnormal auscultatory findings suggestive of pneumonia; rales or evidence of pulmonary consolidation.

Subjects presenting with any of the following would not be considered CAP:

1. Subject had not undergone chest X-ray within 48 hours after admission or that the chest X-ray did not show radiographic findings consistent with pneumonia.
2. Subjects with hospital acquired pneumonia (HAP) which was defined as pneumonia

that occurs ≥ 48h after admission which was not incubating at the time of admission.

3. Subjects were considered to have HAP if they had been hospitalized within the last 30 days prior to admission.

Method to determine incident cases.

1. Database query:

From the regional administrative database, we retrieved data on hospitalizations for which the patient was:

1) adult (aged 18+ years at discharge)

2) residing in Malmö, Svedala or Vellinge at discharge

3) were discharged from a hospital in Skåne during the period 2016-09-18 to 2018-09-18

4) An ICD-10-SE discharge diagnosis code in the primary position of J10.0, J11.0, J12-18, A70.9, A48.1, B01.2, B20.6, J44.0, J44.1, J69.0, J85.1, J85.2, J86.0, and J86.9

The database query returned a list with hospitalizations with patients fulfilling these criteria. We performed manual chart review to determine if the patient fulfilled also the radiological and clinical criteria.

2. Chart review – Radiology:

The hospitalizations retrieved from the database query were screened for x-rays performed within 48 hours of admission. If so, the radiology reports were evaluated for radiographic evidence of pneumonia, defined as the presence of alveolar infiltrates [multilobar, lobar or segmental] containing air bronchograms, e.g. pleural effusion, increased pulmonary density due to infection. All radiology reports were reviewed.

3. Chart review - Clinical symptoms:

Of the patients who met both criteria 1 and 2 described above, we performed chart review to evaluate clinical findings indicating pneumonia. Of the first 200 reviewed patients, 199 (99.5%) showed at least 2 or more symptoms. Therefore, we concluded that chart review regarding clinical symptoms was redundant when the other two criteria were fulfilled, as the clinical criteria were so unspecific and inclusive. Also, as stated in the introduction, the low accessibility to acute hospital beds is associated with a raised admission threshold, i.e. to be admitted you had to fulfil the clinical criteria. Thus, we did not proceed with this time-consuming analysis in more patients.

The results from the database query and chart review will be presented as a flowchart below, fig 1.

Population data

The catchment area of the study was the municipalities of Malmö, Vellinge, and Svedala. The numbers of persons aged over 18 years living in the area, nationally registered and asylum seekers, were collected from the statistical database of Statistics Sweden (15) and the Swedish Migration Board (16). Data was stratified by age in 1-year strata and by sex. The reference time is December 31, hence the data from 2016 and 2017 was used.

Sensitivity analysis – non-primary diagnosis codes

The database was queried once more on adult patients, residing in Malmö Svedala and Vellinge, discharged from a Skåne hospital during the period 2016-09-18 to 2018-09-18 with a CAP diagnosis in a non-primary position, without a CAP diagnosis in the primary position. Of these, we performed a random sample of five patients for each month, resulting in 125 patients. We performed manual chart review for each patient to determine if these patients fulfilled the criteria for CAP as described in the section above, including radiological and clinical criteria.

Comorbidities and outcome

During the chart review, the electronic medical chart system Melior was used to collect information from the medical charts at the time of admission. Information about the age at admission, gender, and comorbidities were collected. The choice of comorbidities was based on the collection of comorbidities made in the previous prospective study in Malmö about pneumonia (OSPIS).

Individual comorbidities are aggregated to at risk and high-risk as mutually exclusive categories.

- (High risk: immunosuppression therapy including systemic corticosteroid use, HIV, AIDS, solid tumor or hematologic malignancies, organ transplantation and chronic kidney disease,
- At risk: COPD, asthma, heart failure, peripheral vascular disease (including stroke and coronary artery disease), diabetes mellitus, chronic liver disease)
- Low risk: For the remaining patients that were not grouped as 'high risk' or at-risk patients.
- In addition, outcome (length-of-stay, ICU transfer and in-hospital mortality) was noted.

In all patients

- demographics: age, sex
- comorbidities: individual comorbidities as well as risk stratification (low risk, at risk, high risk)
- diagnosis: discharge diagnosis in primary position
- outcome: ICU care (interpreted with caution as many patients will be ineligible for ICU referral) and mortality.

Statistical Analysis Plan

Overall incidence rate

The overall crude incidence rate for all-cause CAP will be estimated by dividing the number of incident cases, as defined above, with the person-years at risk from the population data as the denominator. The results will be presented in text as a number with 95% CI per 100.000 person-years. For comparison, age-standardized incidence rate (ASR) will be estimated using the direct standardization method, with the 2013 European standard population as a reference. This result will also be presented as a number with a 95% CI per 100.000 person-years.

Incidence rate, stratified by age and sex.

Incident cases will be categorized into age strata [≥ 18-64, 65-79, 80+], with the corresponding denominator from the population data. For all categories, the age-specific incidence rate with 95% CI will be presented in text. To quantify the relative risk by age strata, standardized incidence ratios (SIR), with 95% CI will be estimated, using the lowest age category as reference. To display the effect of both age and sex on incidence rates, these will be illustrated by a bar plot, as in the example below.

Standardized incidence ratios (SIR)

Standardized Incidence ratio (SIR) will be estimated by applying the incidence rate of the reference category to the category of interest to obtain an expected number of CAP cases. The SIR will then be determined as the ratio between observed CAP cases / expected CAP cases. SIR is used as a risk measure, to obtain the relative risk between groups. If the incidence rate of two groups would be equal the rate would be one. If more cases are observed than in the first group, SIR is greater than one. If fewer cases are observed than in

the first group, SIR is less than one.

Confidence intervals (CI)

Confidence interval will be determined for incidence rates, standardized rates and SIRs, assuming a Poisson distribution. In all estimations, the 95% CI will be used.

Comorbidities and outcome

For descriptive data, median, with interquartile range (IQR), will be used for continuous variables (e.g. age, length of stay). Age will also be categorized in groups ≥ 18-64, 65-79, 80+. For categorical variables, descriptive will use counts (percentages), to describe gender, diagnoses etc. The data will be presented in a table, as in the example below.

Diagnoses

All four-position ICD-10-SE diagnoses will be listed, sorted by frequency, as in the example table below.

Sensitivity analyses

The proportion of incident CAP cases found in the chart review of hospitalizations with a CAP diagnosis in the non-primary position will be extrapolated to the total number of hospitalizations with a CAP diagnosis in the non-primary position. The resulting number will be added to the number of hospitalizations considered as incident cases in the main analysis (with a CAP diagnosis in the primary position). This number will be used to determine a crude incidence rate for the sensitivity analysis, that will be presented in text, as a rate per 100.000 person-years, with 95% CI.

Ethics

The study was conducted in accordance with the Declaration of Helsinki on Ethical Principles for Medical Research Involving Human Subjects, adopted by the General Assembly of the World Medical Association (1996 & 2008). (17) This study was admitted and approved by Lund Regional Ethics Committee (Nos. 2016/220) and Region Skåne's Consultation KVB (kvalitetsregister, vårddatabaser och beredning).

A minor completion was made 2019 for the extra retrospective analysis of medical records. Informed consent was waived for this part and patients were offered an option out by the IRB due to the retrospective nature of the study. All described in the manuscript.

References

1. Heron M. Deaths: leading causes for 2009. Natl Vital Stat Rep. 2012;61(7):1-94.

2. Stralin K, Olcen P, Tornqvist E, Holmberg H. Definite, probable, and possible bacterial aetiologies of community-acquired pneumonia at different CRB-65 scores. Scand J Infect Dis. 2010;42(6-7):426-34.

3. Johansson N, Kalin M, Tiveljung-Lindell A, Giske CG, Hedlund J. Etiology of community-acquired pneumonia: increased microbiological yield with new diagnostic methods. Clin Infect Dis. 2010;50(2):202-9.

4. Welte T. Risk factors and severity scores in hospitalized patients with community-acquired pneumonia: prediction of severity and mortality. Eur J Clin Microbiol Infect Dis. 2012;31(1):33-47.

5. Søgaard M, Nielsen RB, Schønheyder HC, Nørgaard M, Thomsen RW. Nationwide trends in pneumonia hospitalization rates and mortality, Denmark 1997-2011. Respiratory medicine. 2014;108(8):1214-22.

6. Ramirez JA, Wiemken TL, Peyrani P, Arnold FW, Kelley R, Mattingly WA, et al. Adults Hospitalized With Pneumonia in the United States: Incidence, Epidemiology, and Mortality. Clin Infect Dis. 2017;65(11):1806-12.

7. Corrado RE, Lee D, Lucero DE, Varma JK, Vora NM. Burden of Adult Community-acquired, Health-care-Associated, Hospital-Acquired, and Ventilator-Associated Pneumonia: New York City, 2010 to 2014. Chest. 2017;152(5):930-42.

8. Jain S, Self WH, Wunderink RG, Fakhran S, Balk R, Bramley AM, et al. Community-Acquired Pneumonia Requiring Hospitalization among U.S. Adults. N Engl J Med. 2015;373(5):415-27.

9. Kolditz M, Tesch F, Mocke L, Höffken G, Ewig S, Schmitt J. Burden and risk factors of ambulatory or hospitalized CAP: A population based cohort study. Respiratory medicine. 2016;121:32-8.

10. Jokinen C, Heiskanen L, Juvonen H, Kallinen S, Karkola K, Korppi M, et al. Incidence of community-acquired pneumonia in the population of four municipalities in eastern Finland. Am J Epidemiol. 1993;137(9):977-88.

11. Folkhälsomyndigheten. Vaccination programmes and recommendations 2024 [updated 14 December 2022. Available from: https://www.folkhalsomyndigheten.se/the-public-health-agency-of-sweden/communicable-disease-control/vaccinations/vaccination-programmes/.

12. SCB. Kommun i siffror 2020 [Available from: www.scb.se.

13. Malmö stad. Population [Internet]. Malmö: Malmö stad; 2020 [cited 2020 Nov 24]. Available from: https://malmo.se/Fakta-och-statistik/Facts-and-statistics-in-english/Population.html.

14. Welfare N. Klassifikationen ICD-10 Stockholm 2018-12-07 2018. [updated 2020-10-13].

15. Statistics Sweden. 2023 [cited 2023 May 2023]. Available from: https://www.scb.se/en/.

16. SCB Statistics Sweden. Asylum seekers 2002-2023: Swedish Migration Agency; 2024 [cited 2024 May 13]. Available from: https://www.scb.se/en/finding-statistics/statistics-by-subject-area/population/population-composition/population-statistics/pong/tables-and-graphs/asylum-seeker/asylum-seekers/.

17. World Medical Association Declaration of Helsinki: ethical principles for medical research involving human subjects. Jama. 2013;310(20):2191-4.
