# Supplementary material for: The incidence of radiologically verified community-acquired pneumonia requiring hospitalisation in adults living in southern Sweden, 2016-2018: a population-based study
Source: BMC Infect Dis. 2025 Jan 17;25:80. doi: 10.1186/s12879-025-10468-7 (PMC11742510; doi:10.1186/s12879-025-10468-7)
Supplement: Supplementary file 2 — Supplementary Material 2. [file 12879_2025_10468_MOESM2_ESM.docx]

SUPPLEMENTARY APPENDIX

Figure 1. Map of Skåne region

Description of clinical symptoms

Two of the following: Fever (oral temperature (≥38°C) or tympanic temperature (≥38.5°C), hypothermia (≤ 35.5°C), chills or rigors, pleuritic chest pain, cough, sputum production, dyspnea, tachypnea (respiratory rate >20/ min), malaise or abnormal auscultatory findings suggestive of pneumonia; rales or evidence of pulmonary consolidation.

Description of pilot study

From the regional administrative database in Skåne, we retrieved data on adults aged 18+ years, residing in the three municipalities Malmö, Svedala or Vellinge, that were admitted to any of the 10 hospitals in Skåne during the period 18 Sept 2016 to 18 Sept 2018 with a principal discharge diagnosis consistent with pneumonia. The database query returned a list with hospitalizations fulfilling these criteria. We then reviewed 200 patients where 199 (99,5%) showed at least 2 or more of the clinical symtpoms: Fever (oral temperature (≥38°C) or tympanic temperature (≥38.5°C), hypothermia (≤ 35.5°C), chills or rigors, pleuritic chest pain, cough, sputum production, dyspnea, tachypnea (respiratory rate >20/ min), malaise or abnormal auscultatory findings suggestive of pneumonia; rales or evidence of pulmonary consolidation.  The clinical criteria were so comprehensive that almost all patients examined met the described symptoms. One explanation to this is that the threshold för admission today in Sweden is so high (e.g. due to low accessibility of beds) that you need to fulfil clinical symptoms to get admitted. The positive predictive value (PPV) of ICD-10 codes plus radiologic criteria was compared to a gold standard of ICD-10 codes, radiologic criteria plus clinical criteria. Since ICD-10 plus radiologic criteria had 99% accuracy, the case definition used for the final analysis was based on ICD-10 codes and radiological criteria only.

Risk factors for CAP and outcomes

“High risk” patients were immunosuppressive therapy including all types of systemic corticosteroid use, HIV, AIDS, solid tumor or hematologic malignancies, organ transplantation and chronic kidney disease. “At risk” patients were defined as presence of at-risk condition without any high-risk condition: COPD, asthma, heart failure, peripheral vascular disease including stroke and coronary artery disease, diabetes mellitus, chronic liver disease. Finally, “Low risk” patients included individuals that were not classified as “High risk” or “At risk” patients. These categories were based on current ACIP recommendations for pneumococcal vaccination [15]*.*

Included ICD-10-SE discharge diagnosis codes in primary position.

J10.0, Influenza with pneumonia, seasonal influenza virus identified

J11.0, Influenza with pneumonia, virus not identified

J12 Viral pneumonia, not elsewhere classified bronchopneumonia due to viruses other than influenza viruses

J13 Pneumonia due to Streptococcus pneumoniae

J14 Pneumonia due to Haemophilus influenzae

J15 Bacterial pneumonia, not elsewhere classified

J16 Pneumonia due to other infectious organisms, not elsewhere classified

J17 Pneumonia in diseases classified elsewhere

J18 Pneumonia, organism unspecified

A70.9, Chlamydia psittaci infection

A48.1, Legionnaires disease

B01.2, Varicella pneumonia

B20.6, HIV disease resulting in Pneumocystis jirovecii pneumonia

J44.0, Chronic obstructive pulmonary disease with acute lower respiratory infection

J44.1, Chronic obstructive pulmonary disease with acute exacerbation, unspecified

J69.0, Pneumonitis due to food and vomit

J85.1, Abscess of lung with pneumonia

J85.2, Abscess of lung without pneumonia

J86.0, Pyothorax with fistula

J86.9 Pyothorax without fistula
